# Genetic Polymorphisms, Gene–Gene Interactions and Neurologic Sequelae at Two Years Follow-Up in Newborns with Hypoxic-Ischemic Encephalopathy Treated with Hypothermia

**DOI:** 10.3390/antiox10091495

**Published:** 2021-09-20

**Authors:** Katarina Esih, Katja Goričar, Aneta Soltirovska-Šalamon, Vita Dolžan, Zvonka Rener-Primec

**Affiliations:** 1 Division of Pediatrics, Department of Child, Adolescent and Developmental Neurology, University Medical Centre Ljubljana, 1000 Ljubljana, Slovenia; katarina.esih@zd-lj.si; 2 Pharmacogenetics Laboratory, Institute of Biochemistry and Molecular Genetics, Faculty of Medicine, University of Ljubljana, 1000 Ljubljana, Slovenia; katja.goricar@mf.uni-lj.si (K.G.); vita.dolzan@mf.uni-lj.si (V.D.); 3 Division of Pediatrics, Department of Neonatology, University Medical Centre Ljubljana, 1000 Ljubljana, Slovenia; aneta.soltirovska@kclj.si; 4 Department of Pediatrics, Faculty of Medicine, University of Ljubljana, 1000 Ljubljana, Slovenia

**Keywords:** inflammation, oxidative stress, polymorphism, hypoxic-ischemic encephalopathy, newborn

## Abstract

Inflammation and oxidative stress after hypoxic-ischemic brain injury may be modified by genetic variability in addition to therapeutic hypothermia. The aim of our study was to evaluate the association between the polymorphisms in genes of antioxidant and inflammatory pathways in newborns treated with therapeutic hypothermia and the development of epilepsy or CP at two years follow-up. The DNA of 55 subjects was isolated from buccal swabs. Genotyping using competitive allele-specific PCR was performed for polymorphisms in antioxidant (*SOD2* rs4880, *CAT* rs1001179, *GPX1* rs1050450) and inflammatory (*NLRP3* rs35829419, *CARD8* rs2043211, *IL1B* rs1143623, *IL1B* rs16944, *IL1B* rs10716 76, *TNF* rs1800629) pathways. Polymorphic *CARD8* rs2043211 T allele was less frequent in patients with epilepsy, but the association was not statistically significant. The interaction between *CARD8* rs2043211 and *IL1B* rs16944 was associated with epilepsy after HIE: *CARD8* rs2043211 was associated with lower epilepsy risk, but only in carriers of two normal *IL1B* rs16944 alleles (OR_adj_ = 0.03 95% CI = 0.00–0.55; p_adj_ = 0.019). Additionally, *IL1B* rs16944 was associated with higher epilepsy risk only in carriers of at least one polymorphic *CARD8* rs2043211 (OR_adj_ = 13.33 95% CI = 1.07–166.37; p_adj_ = 0.044). Our results suggest that gene–gene interaction in inflammation pathways might contribute to the severity of brain injury in newborns with HIE treated with therapeutic hypothermia.

## 1. Introduction

Perinatal hypoxic-ischemic encephalopathy (HIE) is one of the well-known causes of chronic neurological disability in children, such as epilepsy and/or cerebral palsy (CP) [1].

Therapeutic hypothermia (TH) improves survival after HIE, lowers long-term disability rates, lowers the incidence of epilepsy and CP, lessens the severity of CP [2,3], and reduces the frequency of seizures [4], but it is only partly effective [5]. In newborns with HIE treated with hypothermia epilepsy develops in about 10% [6] and CP in 20% of children [7,8].

Studies propose that TH’s neuroprotective effect is induced by modification of several different molecular pathways [9,10,11,12]. The pathophysiological processes after hypoxic-ischemic (HI) injury include overproduction of reactive oxygen species (ROS) and inflammation [13,14]. The key proteins implicated in these processes are antioxidant enzymes, e.g., superoxide-dismutase (MnSOD), catalase (CAT), and glutathione peroxidase (GPX) [15], inflammasome (NLRP3, CARD8, and caspase-1), and cytokines (interleukin 1β (IL-1β), tumor necrosis factor (TNFα)).

From the clinical point of view, there is a notable interindividual variability in response to TH, which is partially due to the wide variability in hypoxic-ischemic insult between newborns with HIE [16,17]. Until now, studies have shown that the outcome after HIE treated with TH is affected by the severity and duration of the hypoxic-ischemic event [18], Apgar score [3], brain injury pattern detected with magnetic resonance imaging (MRI), and occurrence of neonatal seizures. Neonatal seizures as well as brainstem and deep gray injury on MRI have been shown to be associated with epilepsy development [18,19], and lesions in deep gray matter and the posterior limb of the internal capsule (PLIC) with CP [20].

Genetic factors could also affect the processes of tissue inflammation and destruction following hypoxic-ischemic injury. This could modify the severity of the outcome after HIE and the efficacy of treatment with TH. Common polymorphisms in inflammatory and antioxidant genes that can affect protein activity or gene expression by influencing transcription factor or miRNA binding could therefore influence the risk for long-term complications [21]. Previous studies investigating genetic polymorphisms were mostly focused on the susceptibility for CP after HIE [22,23,24,25,26]. For example, a combination of polymorphisms in *IL1B* and NO synthase 2 (*NOS2*) [26], as well as polymorphisms in oligodendrocyte transcription factor 2 (*OLIG2*) [24], adaptor protein complex 4 (*APC4*) [25], and *CAT* [22] were all associated with the development of CP after HIE. However, several studies do not report if the patients were treated with TH. On the other hand, less is known about genetic factors associated with susceptibility for epilepsy after HIE.

The aim of our study was to evaluate the association between the polymorphisms in genes of antioxidant and inflammatory pathways involved in the pathogenesis after HIE in newborns treated with TH and the development of epilepsy or CP within two years.

## 2. Subjects and Methods

### 2.1. Study Population

Our cross-sectional study included newborns with moderate and severe HIE registered in the electronic database of the neonatal intensive care unit at the University Children’s Hospital Ljubljana. Inclusion criteria were: children born between 2007 and 2019, with ≥36 weeks of gestation and who underwent treatment with TH due to perinatal asphyxia (5 min Apgar score ≤ 5, pH ≤ 7.0, base deficit ≥ 16 mmol/L, or resuscitation 10 min after birth) and neurological signs of moderate to severe encephalopathy [27]. Clinical evidence of moderate or severe encephalopathy was determined by the Sarnat and Sarnat scoring system [28]. Whole-body cooling was started within 6 h after birth and continued for 72 h. After 72 h, the newborns were gradually rewarmed to 36.5 °C.

During TH, all newborns were monitored with amplitude integrated electroencephalography (aEEG). Those with recognized seizures were additionally analyzed with classic EEG and all infants were followed up by classic EEG after rewarming period. The criteria for the diagnosis of neonatal seizures were based on the direct observation of clinical events, confirmed with video EEG and aEEG. Neonatal seizures were defined as sudden, repetitive, evolving, and stereotyped ictal pattern with a clear beginning, middle, and ending and a minimum duration of 5–10 s, with or without clinical event.

The electronic database was checked for all eligible children that could be followed up at the age of 2 years or more. During the years 2018 and 2020, parents or legal guardians of these children received an invitation by telephone call and regular mail, including the description of the study, informed consent, and buccal swab with instructions to obtain a sample for DNA extraction. The recruitment of the samples was completed in 2020.

The diagnosis of epilepsy and CP was documented. Epilepsy was defined according to the International League Against Epilepsy (ILAE) 2014 as at least 2 unprovoked seizures at least 24 h apart; one unprovoked seizure and a probability of further seizures similar to the general recurrence risk after two unprovoked seizures, occurring over the next 10 years; diagnosis of an epilepsy syndrome [29].

CP was defined as a group of disorders of development and posture, causing activity limitation, that are attributed to non-progressive disturbances that occurred in the developing infant or fetal brain [30]. It was classified by motor type and gross motor function using the Gross Motor Function Classification System (GMFCS) [31].

Among 84 children with HIE who were treated with total body hypothermia, 29 were excluded due to the following reasons: the informed consent was not provided, death of the patient, rejection by parents or legal guardians to participate in the study already by telephone call, age < 2 years, or insufficient contact data availability.

In a subgroup of 43 newborns, magnetic resonance imaging (MRI) was performed within the first week (between 4th and 7th days).

The study was approved by the Republic of Slovenia National Medical Ethics Committee (0120-303/2015/9, dated 11 April 2018) and informed consent was obtained from all the participants’ parents or legal guardians before inclusion in the study.

### 2.2. MR Scoring System

The MRI was conducted in line with our previously described protocol [32]. The patterns of brain injury were classified according to the Rutherford classification [33]. The pattern and severity of injury in the 4 regions were evaluated and scored: posterior limb of internal capsule (PLIC), thalamus and basal ganglia, white matter, and cortex, as previously described in the literature [33,34,35]. Additionally, the pattern and severity of brainstem injury were analyzed, i.e., lesions at mesencephalon and in pons. An overall assessment was determined by adding up all 5 regional subscores, and classified as normal, mild, moderate, and severe injury [36]. Subjects were classified as normal if there was no injury seen on MRI. If there were mild, moderate or severe MRI findings in one, two or all of the assessed regions, overall assessment was defined as mild, moderate or severe, respectively. For all analyses, we compared subjects with normal/mild to subjects with moderate/severe pathological MRI findings.

### 2.3. DNA Extraction and Genotyping

For all children, buccal swabs were obtained for the extraction of genomic DNA using QIA amp DNA Mini Kit (Qiagen, Hilden, Germany) according to the manufacturer’s protocol. Single-nucleotide polymorphisms (SNPs) with minor allele frequency above 5% in the European population and experimentally confirmed or in silico predicted function in antioxidant (*SOD2* rs4880, *CAT* rs1001179, *GPX1* rs1050450) and inflammatory (*NLRP3* rs35829419, *CARD8* rs2043211, *IL1B* rs1143623, *IL1B* rs16944, *IL1B* rs1071676, *TNF* rs1800629) pathways were included in the study. Genotyping was performed using a fluorescent-based, competitive, allele-specific polymerase chain reaction (KASP, LGC Genomics, Hoddesdon, UK) according to the manufacturer’s protocol.

### 2.4. Statistical Analysis

Median with interquartile range (25–75%) and frequencies were used to describe continuous and categorical variables, respectively. Deviation from the Hardy–Weinberg equilibrium (HWE) was calculated using the standard χ^2^ test. Dominant genetic model was used in all analyses. For *IL1B* rs16944, the more frequent polymorphic C allele was used as the reference allele in all analyses. Logistic regression was used to evaluate the association of investigated SNPs with CP or epilepsy using odds ratios (ORs) and 95% confidence intervals (CIs). Fisher’s exact test was used if there were no patients within one of the categories. Multiplicative gene–gene interactions were assessed using logistic regression. To evaluate the combined effect of all *IL1B* SNPs, Thesias program was used for haplotype analysis [37]. The most common haplotype was used as a reference in all analyses.

As nine SNPs were investigated, Bonferroni correction was used to account for multiple comparisons: *p*-values below 0.006 were considered statistically significant, while *p*-values between 0.006 and 0.050 were considered nominally significant. All statistical tests were two-sided. For a polymorphism with minor allele frequency of 0.30, this study had 80% power to detect ORs of 5.5 or more. Power calculation was conducted by the PS Power and sample size calculations, version 3.0 [38]. The statistical analysis was performed using IBM SPSS Statistics version 27.0 (IBM Corporation, Armonk, NY, USA).

## 3. Results

In total, 55 newborns met the inclusion criteria. Clinical characteristics of the children are presented in Table 1. At two years follow up, 16 patients developed epilepsy (29.1%) and 15 children CP (27.3%). Further, both conditions were present in 13 (24%) children. The most frequent motor type of CP was spastic CP (11, 73.3%) and grade 5 (8; 57.1%) according to the GMCSF score.

Table 2 represents the association of clinical characteristics of patients with epilepsy and CP. Among the clinical parameters, only neonatal convulsions were significantly associated with increased risk for the development of both epilepsy and CP (*p* = 0.007 and *p* = 0.012, respectively). Delivery using caesarean section was compared with vaginal delivery including vacuum extraction. Epilepsy and CP were less common in caesarean section, but the difference was not significant (*p* = 0.055 and *p* = 0.216, respectively). In a subgroup of 43 patients where MRI was performed, 12 (28%) patients had epilepsy and nine (21%) had CP. The moderate–severe brain injury MRI pattern was present in 17 (39.5%) patients and it was significantly associated with an increased risk for the development of epilepsy and CP (*p* = 0.001 and *p* < 0.001, respectively) (Table 3).

### 3.1. Association of Polymorphisms in Antioxidant and Inflammatory Pathways with Epilepsy and CP

The genotype frequencies and minor allele frequencies of polymorphisms in the antioxidant and inflammatory pathways are presented in Supplemental Table S1. All of the investigated polymorphisms were in Hardy–Weinberg equilibrium (Supplemental Table S1).

Polymorphic *CARD8* rs2043211 T allele was less frequent in patients with epilepsy, however the association was not statistically significant, even after adjustment for neonatal convulsions (*p* = 0.207 and *p* = 0.070, respectively). None of the other polymorphisms were associated with epilepsy in univariable or multivariable analysis (Table 4). Additionally, none of the studied polymorphisms were associated with the development CP in univariable analysis or after adjustment for neonatal convulsions (Table 5).

As three *IL1B* polymorphisms were included in our study, we additionally analyzed the haplotypes of *IL1B*: seven haplotypes were predicted in our cohort, however only four had frequencies above 5% (GCC, GCG, CTC, and CTG) (Supplemental Table S2). The CTG haplotype carrying less frequent *IL1B* rs1143623 C and *IL1B* rs16944 T alleles was more frequent in patients with epilepsy (OR =2.51 95% CI = 0.70–8.96; *p* = 0.156) and CP (OR =3.53 95% CI = 0.89–13.99; *p* = 0.072) compared to the reference GCC haplotype, but the difference did not reach statistical significance.

### 3.2. Association of Gene-gene Interactions with Epilepsy and CP

*CARD8* and *IL1B* polymorphisms were previously associated with MRI brain injury patterns [32], that were important predictors of epilepsy and CP in our study. We therefore examined the interactions between *CARD8* and *IL1B* polymorphisms and epilepsy or CP. After adjustment for neonatal convulsions, we observed a nominally significant interaction between *CARD8* rs2043211 and *IL1B* rs16944 and epilepsy risk (*p* = 0.033; Table 6). Carriers of at least one polymorphic *CARD8* rs2043211 allele were less likely to develop epilepsy only in carriers of two normal *IL1B* rs16944 alleles (OR_adj_ = 0.03 95% CI = 0.00–0.55; p_adj_ = 0.019). Similarly, carriers of at least one polymorphic *IL1B* rs16944 allele were more likely to develop epilepsy only in carriers of at least one polymorphic *CARD8* rs2043211 (OR_adj_ = 13.33 95% CI = 1.07–166.37; p_adj_ = 0.044). Similar, although not significant trend was observed for the interaction between *CARD8* rs2043211 and *IL1B* rs1143623 and epilepsy risk (*p* = 0.051; Table 6). Carriers of at least one polymorphic *CARD8* rs2043211 allele were less likely to develop epilepsy only in carriers of two normal *IL1B* rs1143623 alleles (OR_adj_ = 0.05 95% CI = 0.00–0.69; p_adj_ = 0.025).

The interaction between *CARD8* rs2043211 and *IL1B* rs16944 and CP risk was not significant (*p* = 0.073; Table 6). Still, carriers of at least one polymorphic *CARD8* rs2043211 allele were less likely to develop CP only in carriers of two normal *IL1B* rs16944 alleles (OR_adj_ = 0.07 95% CI = 0.01–0.99; p_adj_ = 0.049). Carriers of at least one polymorphic *IL1B* rs16944 allele were more likely to develop CP only in carriers of at least one polymorphic *CARD8* rs2043211 (OR_adj_ = 13.33 95% CI = 1.07–166.37; p_adj_ = 0.044).

## 4. Discussion

Our present study investigated the association of the common polymorphisms in antioxidant and inflammatory genes, haplotypes and gene–gene interactions with development of epilepsy and CP after perinatal HIE treated with TH. The most important finding was that the interaction between *CARD8* rs2043211 and *IL1B* rs16944 was associated with epilepsy after HIE. *CARD8* rs2043211 was associated with lower epilepsy risk, while *IL1B* rs16944 was associated with higher epilepsy risk, (when stratified for *IL1B* rs16944 or *CARD8* rs2043211 genotype, respectively).

In our study group, 29.1% patients developed epilepsy and 27.3% developed CP. A similar incidence for CP was observed in other studies, as reported in the Cochrane meta-analysis, where 23% of patients developed CP after HIE treated with whole-body cooling TH [39]. However, in the Cochrane meta-analysis, the incidence of epilepsy was not observed. Instead, they reported the effect of TH on the presence of seizures or need for anticonvulsant treatment on follow up, which was 11%. The high incidence of epilepsy in our cohort of children might be explained by large number of newborns (65%) with moderate to severe brain injury pattern on MRI, mostly brainstem and deep gray injury which have been shown to be associated with epilepsy later [15,18]. However, due to different outcomes used in different studies, the results regarding epilepsy are not directly comparable.

Among the clinical parameters, only neonatal convulsions were significantly associated with the development of epilepsy and CP in our study, consistent with what was previously reported in other studies [40,41]. The moderate–severe brain injury pattern on MRI was also significantly associated with the development of epilepsy and CP. The early MRI results were already demonstrated to be good predictors of clinical outcome [42].

In our study, *CARD8* rs2043211 T allele was associated with a lower frequency of epilepsy after HIE after the adjustment for neonatal convulsion, but its association was nominally significant only in interaction with *IL1B* rs16944 after adjustment for neonatal convulsions. Consistent with these results, in our previous study, *CARD8* rs2043211 T allele was associated with lower overall rates of brain injury according to the MRI brain injury patterns after HIE in newborns treated with TH [32]. CARD8 is a component of a multiprotein complex inflammasome, which plays an important role in central nervous system inflammation [43,44]. Dysregulated inflammation is one of the main contributors of brain tissue damage. During hypoxic-ischemic insult, inflammatory cells such as astrocytes and microglia are activated and secrete inflammatory mediators that may worsen the tissue damage [45].

The functional role of *CARD8* rs2043211 is not fully understood. In previous studies, rs2043211 was associated with a protective role in various conditions, also non-neurological [46,47,48,49]. Contrary to these findings, some other studies reported its deleterious effect in other diseases [50,51]. As a result of alternative splicing, *CARD8* has five known mRNA isoforms and the function of rs2043211 differs among transcripts: it either introduces a stop codon or leads to amino acid change [44]. The functional role of these isoforms is not clear, but it could help explain the contradictory results observed in previous studies [44]. Additionally, the role of *CARD8* might vary in different pathologies in different organs.

In our study, carriers of at least one polymorphic *CARD8* rs2043211 allele were less likely to develop epilepsy only in carriers of two normal *IL1B* rs16944 or rs1143623 alleles. CARD8 functions as a negative regulator of inflammasome and prevents its assembly. Consequently, it blocks the release of IL-1β [52], which could explain the observed gene–gene interaction.

Carriers of at least one polymorphic *IL1B* rs16944 allele were more likely to develop epilepsy only when carrying at least one polymorphic *CARD8* rs2043211, further emphasizing the important role of *CARD8*-*IL1B* gene–gene interaction. In our previous study, *IL1B* rs16944 was associated with an increased risk of posterior limb of internal capsule (PLIC) injury [32]. Additionally, *IL1B* rs16944 was associated with development of CP after HIE in a previous study [26], consistent with our results, and also various other diseases, including mental disorders [53,54]. Promotor *IL1B* rs16944 polymorphism can affect transcription factor binding and transcription of the *IL1B* gene [26]. It was associated with increased IL-1β secretion [55] and higher serum IL-1β levels [56]. We could thus speculate that a higher inflammatory response might be associated with poor neurologic outcome after HIE.

However, in haplotype analysis, *IL1B* haplotypes were not significantly associated with epilepsy or CP, even though the CTG haplotype carrying *IL1B* rs1143623 and *IL1B* rs16944 alleles was more frequent in patients with epilepsy. This suggests that interaction with inflammasome may be more important regarding the risk of neurological disability than genetic variability of individual genes. Previously, only gene–gene interactions between *IL1B* and *TNF* were investigated, but no association with epilepsy was observed when comparing patients with epilepsy and healthy controls [57].

In the present study, polymorphisms in antioxidant genes were not associated with neurological outcome. Previously, *CAT* rs1001179 was found to be associated with CP only in children with HIE that were not treated with TH [22], suggesting the role of genetic variability in antioxidant genes might differ in response to TH.

To the best of our knowledge, our study was the first to evaluate the role of genetic variability and the risk of epilepsy after HIE in newborns treated with TH. Some studies have already been performed regarding polymorphisms and the risk of epilepsy [23,57,58,59], but none of them investigated epilepsy after HIE treated with TH separately from other epilepsy causes.

Furthermore, the strength of our study is that our study group was homogenous as we only included term newborns after HIE treated with TH. We were also able to compare the clinical outcome and the MRI results from our previous study. We additionally characterized several polymorphisms in the inflammatory and antioxidant pathways and their role in HIE and studied their haplotypes and gene–gene interactions.

However, the limitation of our study was the nature of the study design and small study sample. Due to the cross-sectional design, we were not able to obtain the MRI results for all patients included. Furthermore, we also had to exclude the patients who did not reach the age of two years when clinical evaluation was performed. Further studies are therefore needed to clarify the role of gene–gene interactions in inflammatory pathways and outcome after HIE.

## 5. Conclusions

In conclusion, this is the first study that demonstrated that gene–gene interactions in inflammatory pathways may contribute to the clinical outcome after HIE in newborns treated with TH. Gene–gene interactions may play a more important role in response to HIE than individual genetic polymorphisms. Our results could contribute to better understanding of the long-term neurological outcome in these patients.

## Figures and Tables

**Table 1:** Clinical characteristics of 55 newborns with HIE.

Clinical Characteristic	Category	N (%)
Sex	Male	33 (60.0)
Mode of delivery	Vaginal	16 (31.4) [4]
	Caesarean section	31 (60.8)
	Vacuum extraction	4 (7.8)
Apgar score in 5 min	≤5	40 (81.6) [6]
	>5	9 (18.4)
Apgar score in 10 min	≤5	21 (46.7) [10]
	>5	24 (53.3)
Sarnat and Sarnat score	2	25 (48.1) [3]
	3	27 (51.9)
Neonatal convulsions	No	23 (43.4) [2]
	Yes	30 (55.6)
Epilepsy	No	39 (70.9)
	Yes	16 (29.1)
Cerebral palsy	No	40 (72.7)
	Yes	15 (27.3)
GMCSF (1–5)	1	1 (7.1) [1]
	2	1 (7.1)
	3	2 (14.3)
	4	2 (14.3)
	5	8 (57.1)
Cerebral palsy type	Spastic	11 (73.3)
	Dystonic	2 (13.3)
	Spastic-dystonic	2 (13.3)
**Clinical Characteristic**	**Unit**	**Median (25–75%)**
Gestational age	weeks	39 (38–40) [2]
Birthweight	g	3300 (2793–3500) [5]
Head circumference	cm	34.8 (33–35.5) [11]

Number of subjects with missing data is presented in [] brackets.

**Table 2:** Clinical characteristics of patients with epilepsy and cerebral palsy (CP).

Characteristic		Epilepsy N (%)	OR (95% CI)	*p*-Value	CP N (%)	OR (95% CI)	*p*-Value
Gender	Male	10 (30.3)	Ref.		9 (27.3)	Ref.	
	Female	6 (27.3)	0.86 (0.26–2.85)	0.809	6 (27.3)	1.00 (0.30–3.36)	1.000
Mode of delivery	Vaginal/vacuum	9 (45.0)	Ref.		7 (35.0)	Ref.	
	Caesarean section	6 (19.4)	0.29 (0.08–1.03)	0.055	6 (19.4)	0.45 (0.12–1.60)	0.216
Apgar score in 5 min	>5	3 (33.3)	Ref.		2 (22.2)	Ref.	
	≤5	12 (30.0)	0.86 (0.18–4.01)	0.845	12 (30.0)	1.50 (0.27–8.30)	0.642
Apgar score in 10 min	>5	4 (16.7)	Ref.		4 (16.7)	Ref.	
	≤5	8 (38.1)	3.08 (0.77–12.34)	0.113	8 (38.1)	3.08 (0.77–12.34)	0.113
Sarnat and Sarnat grading	2	7 (28.0)	Ref.		5 (20.0)	Ref.	
	3	9 (33.3)	1.29 (0.39–4.20)	0.677	10 (37.0)	2.35 (0.67–8.24)	0.181
Neonatal convulsions	No, N (%)	2 (8.7)	Ref.		2 (8.7)	Ref.	
	Yes, N (%)	14 (46.7)	9.19 (1.82–46.34)	0.007	13 (43.3)	8.03 (1.59–40.58)	0.012

Legend: CI = confidence interval, OR = odds ratio.

**Table 3:** The association of category of MRI brain pattern with epilepsy and cerebral palsy (CP).

MRI Brain Pattern Overall Assessment	Without Epilepsy N (%)	With Epilepsy N (%)	OR (95% CI)	*p*-Value	without CP N (%)	with CP N (%)	*p*-Value
Normal + mild	25 (96.2)	1 (3.8)	Ref.		26 (100.0)	0 (0.0)	
Moderate + severe	6 (35.3)	11 (64.7)	45.83 (4.92–427.36)	0.001	8 (47.1)	9 (52.9)	<0.001 *

* calculated using Fisher’s exact test. Legend: CI = confidence interval, OR = odds ratio.

**Table 4:** The association of common polymorphisms with epilepsy.

SNP	Genotype	without Epilepsy N (%)	with Epilepsy N (%)	OR (95% CI)	*p*	OR (95% CI) Adj	P_adj_
*SOD2* rs4880	CC	12 (70.6)	5 (29.4)	Ref.		Ref.	
	CT + TT	27 (71.1)	11 (28.9)	0.98 (0.28–3.44)	0.972	1.1 (0.27–4.4)	0.894
*CAT* rs1001179	CC	24 (64.9)	13 (35.1)	Ref.		Ref.	
	CT + TT	15 (83.3)	3 (16.7)	0.37 (0.09–1.51)	0.166	0.36 (0.08–1.65)	0.187
*GPX1* rs1050450	CC	17 (68)	8 (32)	Ref.		Ref.	
	CT + TT	22 (73.3)	8 (26.7)	0.77 (0.24–2.48)	0.665	1.07 (0.29–3.95)	0.914
*NLRP3* rs35829419	CC	34 (72.3)	13 (27.7)	Ref.		Ref.	
	CA	5 (62.5)	3 (37.5)	1.57 (0.33–7.52)	0.573	1.38 (0.24–7.76)	0.717
*CARD8* rs2043211	AA	17 (63)	10 (37)	Ref.		Ref.	
	AT + TT	22 (78.6)	6 (21.4)	0.46 (0.14–1.53)	0.207	0.27 (0.07–1.11)	0.070
*IL1B* rs1143623	GG	21 (75)	7 (25)	Ref.		Ref.	
	GC + CC	18 (66.7)	9 (33.3)	1.50 (0.46–4.84)	0.497	1.86 (0.5–6.92)	0.352
*IL1B* rs16944	CC	21 (77.8)	6 (22.2)	Ref.		Ref.	
	TC + TT	18 (64.3)	10 (35.7)	1.94 (0.59–6.40)	0.274	1.99 (0.54–7.42)	0.303
*IL1B* rs1071676	GG	14 (63.6)	8 (36.4)	Ref.		Ref.	
	GC + CC	25 (75.8)	8 (24.2)	0.56 (0.17–1.82)	0.335	0.58 (0.16–2.13)	0.411
*TNF* rs1800629	GG	27 (73)	10 (27)	Ref.		Ref.	
	GA + AA	12 (66.7)	6 (33.3)	1.35 (0.40–4.57)	0.630	1.18 (0.31–4.54)	0.811

Adj: adjusted for neonatal convulsions. Legend: CI = confidence interval, OR = odds ratio, SNP = single-nucleotide polymorphism.

**Table 5:** The association of common polymorphisms with cerebral palsy (CP).

SNP	Genotype	without CP N (%)	with CP N (%)	OR (95% CI)	*p*	OR (95% CI) Adj	P_adj_
*SOD2* rs4880	CC	11 (64.7)	6 (35.3)	Ref.		Ref.	
	CT + TT	29 (76.3)	9 (23.7)	0.57 (0.16–1.97)	0.374	0.57 (0.14–2.24)	0.419
*CAT* rs1001179	CC	25 (67.6)	12 (32.4)	Ref.		Ref.	
	CT + TT	15 (83.3)	3 (16.7)	0.42 (0.10–1.72)	0.226	0.42 (0.09–1.91)	0.262
*GPX1* rs1050450	CC	18 (72)	7 (28)	Ref.		Ref.	
	CT + TT	22 (73.3)	8 (26.7)	0.94 (0.28–3.07)	0.912	1.33 (0.35–4.96)	0.675
*NLRP3* rs35829419	CC	34 (72.3)	13 (27.7)	Ref.		Ref.	
	CA	6 (75)	2 (25)	0.87 (0.16–4.88)	0.876	0.7 (0.11–4.39)	0.701
*CARD8* rs2043211	AA	18 (66.7)	9 (33.3)	Ref.		Ref.	
	AT + TT	22 (78.6)	6 (21.4)	0.55 (0.16–1.82)	0.325	0.36 (0.09–1.41)	0.143
*IL1B* rs1143623	GG	22 (78.6)	6 (21.4)	Ref.		Ref.	
	GC + CC	18 (66.7)	9 (33.3)	1.83 (0.55–6.13)	0.325	2.32 (0.61–8.84)	0.216
*IL1B* rs16944	CC	22 (81.5)	5 (18.5)	Ref.		Ref.	
	TC + TT	18 (64.3)	10 (35.7)	2.44 (0.71–8.46)	0.158	2.58 (0.67–9.94)	0.169
*IL1B* rs1071676	GG	15 (68.2)	7 (31.8)	Ref.		Ref.	
	GC + CC	25 (75.8)	8 (24.2)	0.69 (0.21–2.28)	0.538	0.74 (0.2–2.74)	0.653
*TNF* rs1800629	GG	29 (78.4)	8 (21.6)	Ref.		Ref.	
	GA + AA	11 (61.1)	7 (38.9)	2.31 (0.67–7.88)	0.183	2.25 (0.58–8.7)	0.238

Adj: adjusted for neonatal convulsions. Legend: CI = confidence interval, OR = odds ratio, SNP = single-nucleotide polymorphism.

**Table 6:** The interaction of *CARD8* and *ILB* polymorphisms with epilepsy or cerebral palsy.

	Epilepsy				CP			
Interaction	OR (95% CI)	*p*	OR (95% CI) adj	P_adj_	OR (95% CI)	*p*	OR (95% CI) adj	P_adj_
*CARD8* rs2043211 & *IL1B* rs1143623	5.00 (0.42–59.16)	0.202	20.20 (0.99–412.40)	0.051	3.50 (0.29–42.46)	0.325	9.93 (0.56–175.78)	0.117
*CARD8* rs2043211 & *IL1B* rs16944	12.50 (0.76–205.33)	0.077	32.91 (1.33–811.49)	0.033	8.75 (0.52–146.93)	0.132	16.96 (0.77–375.99)	0.073
*CARD8* rs2043211 & *IL1B* rs1071676	0.90 (0.07–11.43)	0.937	0.47 (0.03–8.21)	0.608	2.80 (0.23–33.93)	0.419	1.85 (0.12–28.78)	0.660

Adj: adjusted for neonatal convulsions. Legend: CI = confidence interval, OR = odds ratio.

## Data Availability

All the data is presented within the article and in Supplementary Material. Any additional information is available on request from the corresponding author.

## Supplementary Materials

The following are available online at https://www.mdpi.com/article/10.3390/antiox10091495/s1, Table S1: The association of *IL1B* haplotypes with epilepsy and cerebral palsy (CP), Table S2: Genotype frequencies.
