# Supplementary material for: Genetic Polymorphisms, Gene–Gene Interactions and Neurologic Sequelae at Two Years Follow-Up in Newborns with Hypoxic-Ischemic Encephalopathy Treated with Hypothermia
Source: Antioxidants (Basel). 2021 Sep 20;10(9):1495. doi: 10.3390/antiox10091495 (PMC8465839; doi:10.3390/antiox10091495)
Supplement: Supplementary file 1 [file antioxidants-10-01495-s001.zip › antioxidants-1323980-supplementary.pdf]

Supplementary Materials:

Table S1: The association of *IL1B* haplotypes with epilepsy and cerebral palsy (CP)

**Haplotype**	**Estimated frequency**		**OR (95% CI)**	**P**	**Estimated frequency**		**OR (95% CI)**	**P**
	**Without epilepsy**	**With epilepsy**			**Without CP**	**CP**		
GCC	0.38	0.33	Ref.		0.402	0.276	Ref.	
GCG	0.31	0.27	1.17 (0.38-3.57)	0.788	0.298	0.290	1.61 (0.48-5.38)	0.438
CTC	0.17	0.33	2.51 (0.70-8.96)	0.156	0.164	0.357	3.53 (0.89-13.99)	0.072
CTG	0.09	0.01	0.85 (0.00-19583.92)	0.975	0.086	0.010	0.94 (0.00-575325.17)	0.993

The SNPs are ordered from the 5'- to 3'-end as follows: rs1143623, rs16944, and rs1071676

Table S2: Genotype frequencies

**Gene**	**SNP**	**Role**	**Genotype**	**N (%)**	**MAF**	**pHWE**
SOD2	rs4880	p.Ala16Val	CC	17 (30.9)	0.400	0.116
			CT	32 (58.2)		
			TT	6 (10.9)		
CAT	rs1001179	c.-262C>T	CC	37 (67.3)	0.173	0.545
			CT	17 (30.9)		
			TT	1 (1.8)		
GPX1	rs1050450	p.Pro198Leu	CC	25 (45.5)	0.336	0.639
			CT	23 (41.8)		
			TT	7 (12.7)		
NLRP3	rs35829419	p.Gln705Lys	CC	47 (85.5)	0.073	0.561
			CA	8 (14.5)		
CARD8	rs2043211	p.Cys10Ter	AA	27 (49.1)	0.309	0.639
			AT	22 (40.0)		
			TT	6 (10.9)		
IL1B	rs1143623	c.-1560G>C	GG	28 (50.9)	0.291	0.821
			GC	22 (40.0)		
			CC	5 (9.1)		
	rs16944	c.-598T>C	TT	8 (14.5)	0.673[a]	0.196
			TC	20 (36.4)		
			CC	27 (49.1)		
	rs1071676	c.*505G>C	GG	22 (40.0)	0.373	0.836
			GC	25 (45.5)		
			CC	8 (14.5)		
TNF	rs1800629	c.-308G>A	GG	37 (67.3)	0.191	0.385
			GA	15 (27.3)		
			AA	3 (5.5)		

[a]variant allele frequency
